# Neuroendocrine Changes in Cholangiocarcinoma Growth

**DOI:** 10.3390/cells9020436

**Published:** 2020-02-13

**Authors:** Keisaku Sato, Heather Francis, Tianhao Zhou, Fanyin Meng, Lindsey Kennedy, Burcin Ekser, Leonardo Baiocchi, Paolo Onori, Romina Mancinelli, Eugenio Gaudio, Antonio Franchitto, Shannon Glaser, Gianfranco Alpini

**Affiliations:** 1 Division of Gastroenterology and Hepatology, Department of Medicine, Indiana University School of Medicine, Indianapolis, IN 46202, USA; 2 Richard L. Roudebush VA Medical Center, Indianapolis, IN 46202, USA; 3 Department of Medical Physiology, Texas A&M University College of Medicine, Bryan, TX 77807, USA; 4 Division of Transplant Surgery, Department of Surgery, Indiana University School of Medicine, Indianapolis, IN 46202, USA; 5 Liver Unit, Department of Medicine, University of Rome “Tor Vergata”, 00133 Rome, Italy; 6 Department of Anatomical, Histological, Forensic Medicine and Orthopedics Sciences, Sapienza University of Rome, 00185 Rome, Italy; 7 Eleonora Lorillard Spencer Cenci Foundation, 00185 Rome, Italy

**Keywords:** cholangiocarcinoma, cholangiocytes, ductular reaction, liver fibrosis, neurotransmitters, neuropeptides, hormones

## Abstract

Cholangiocarcinoma (CCA) is a highly aggressive malignancy that emerges from the biliary tree. There are three major classes of CCA—intrahepatic, hilar (perihilar), or distal (extrahepatic)—according to the location of tumor development. Although CCA tumors are mainly derived from biliary epithelia (i.e., cholangiocytes), CCA can be originated from other cells, such as hepatic progenitor cells and hepatocytes. This heterogeneity of CCA may be responsible for poor survival rates of patients, limited effects of chemotherapy and radiotherapy, and the lack of treatment options and novel therapies. Previous studies have identified a number of neuroendocrine mediators, such as hormones, neuropeptides, and neurotransmitters, as well as corresponding receptors. The mediator/receptor signaling pathways play a vital role in cholangiocyte proliferation, as well as CCA progression and metastases. Agonists or antagonists for candidate pathways may lead to the development of novel therapies for CCA patients. However, effects of mediators may differ between healthy or cancerous cholangiocytes, or between different subtypes of receptors. This review summarizes current understandings of neuroendocrine mediators and their functional roles in CCA.

## 1. Introduction

Cholangiocarcinoma (CCA) is a biliary tract cancer with multiple liver cell involvement, which is highly malignant [1]. CCA is one of the most fatal cancers because early diagnosis is challenging, and treatment options are scarce [1]. Treatments for CCA include chemotherapy and radiotherapy, liver transplantation, or surgical resection [1,2]. However, patients with curative surgeries often have recurrence and distant malignancies, and chemotherapy has only limited effects due to genetic mutations or aberrations resulting in chemoresistance [2]. As a result, the five year survival rate is less than 30% [3,4]. CCA is the second most common primary hepatic malignancy after hepatocellular carcinoma (HCC) and accounts for approximately 20% of deaths from hepatobiliary cancers [3]. In addition, the incidence of CCA has been increasing in recent years probably due to the increased incidence of metabolic syndromes and obesity, which are risk factors for CCA development [5].

CCA is heterogeneous and can be classified depending on the location where the tumor emerges: intrahepatic, hilar or perihilar, or distal or extrahepatic CCA [6]. The origins of CCA tumors can also be heterogeneous. The majority of CCA tumors, especially for hilar and distal CCA, are derived from the bile duct epithelia (i.e., cholangiocytes). In rare cases of intrahepatic CCA, liver stem cells or hepatic progenitor cells behave as cancer stem cells and develop as CCA tumors [7,8] In addition, hepatocytes can transdifferentiate into cancerous biliary phenotypes in rodents, indicating that intrahepatic CCA tumors can be derived from hepatocytes [9,10]. These heterogeneous characteristics may be responsible for poor survival rates of patients and limited effects of current therapies such as chemotherapy.

Primary sclerosing cholangitis (PSC) is a type of cholangiopathy, which is characterized by cholestasis, biliary inflammation, ductular reaction, and liver fibrosis [11]. It is known that PSC patients have high risks of CCA development, and a previous study using 211 PSC patients reported that the risk of CCA after 10 or 20 years was both 9%, indicating the strong association between PSC and CCA [12]. Although detailed mechanisms of the development of CCA tumors are undefined, previous studies have identified a number of neurotransmitters, hormones, and peptides that are associated with cholangiocyte functions in PSC as well as CCA development. These associated mediators and their receptors may have the potential as novel therapeutic targets to inhibit CCA development or progression. This review summarizes current understandings of neuroendocrine changes in the liver associated with CCA.

## 2. Functional Roles of Neuroendocrine Mediators in Cholangiocarcinoma

### 2.1. Hormones

#### 2.1.1. Secretin

Secretin (Sct) is a hormone produced predominantly by S cells of the duodenum [13]. Cholangiocytes also secrete Sct and express its receptor (secretin receptor, SR), which is upregulated when cells are activated [14]. Sct induces cholangiocyte proliferation via elevated cAMP levels leading to ductular reaction in healthy rats in vivo [15]. Cholangiocyte activation and proliferation is mediated by decreased levels of microRNA (miRNA) let-7a and miR-125b as well as elevated levels of their targets, nerve growth factor (NGF) and vascular endothelial growth factor (VEGF), and the Sct/SR axis is essential in this process [16]. Bile duct ligation (BDL), which is a surgical procedure to mimic cholestasis in rodents, causes ductular reaction, liver inflammation, and fibrosis [17]. Knocking out of Sct or SR improved liver conditions of BDL mice by inhibiting activation and fibrogenesis in hepatic stellate cells (HSCs) [18]. Transgenic mice with knockout of multidrug resistance protein 2 (*Mdr2*^-/-^ mice) are the most common transgenic animal model of human PSC [17]. Depletion of SR in *Mdr2*^-/-^ mice attenuated ductular reaction and liver fibrosis by decreasing cellular senescence in cholangiocytes in vivo [19]. The Sct/SR axis is a promising therapeutic target to regulate cholangiocyte functions followed by liver fibrosis [18,20]. SR is expressed only in cholangiocytes in the liver [21,22]. CCA tumors have biliary phenotypes and are positive for biliary markers such as cytokeratin-7 (CK-7) and CK-19 in immunohistochemistry [23]. If the origin of CCA tumors is cholangiocytes, these tumors are also positive for SR. A previous study analyzed 10 cirrhotic liver tissues, 35 CCA tumors, and 45 HCC tumors and found that SR was upregulated with ductular reaction in cirrhotic livers [24]. Sixty-three percent of CCA tumors were positive to SR, but no HCC tumors expressed SR [24]. These studies support that HCC tumors do not express SR and suggest that the majority of CCA tumors are cholangiocyte-derived (63% in this study), but there are other phenotypes that are derived from different hepatic cells and do not express SR. Although Sct induces cell proliferation in normal cholangiocytes, it has opposite effects on CCA. Sct treatment decreased cell proliferation in human CCA cell lines, Mz-ChA-1, SG231, HuH-28, CCLP, HuCCT1, and TFK-1 cells, but increased proliferation of normal human cholangiocytes in vitro [25]. Sct elevated cAMP levels in normal cholangiocytes but not in Mz-ChA-1 cells, and Sct administration decreased tumor size in Mz-ChA-1 xenograft mice in vivo [25]. Although the Sct/SR axis may be useful as a therapeutic target for CCA using Sct administration or SR agonists, Sct production is upregulated in PSC cholangiocytes and it is unclear how CCA tumors emerge in this condition. In addition, since the limited percentages of CCA tumors are positive for SR, the efficacy of Sct treatment will also be limited only to SR-positive CCA patients.

#### 2.1.2. Somatostatin

Somatostatin is a hormone secreted from various organs including the brain, the pancreas, and the gut [26]. Somatostatin functions as an inhibitory hormone which regulates cell proliferation and secretion of hormones in various types of cells [26]. Cholangiocytes express receptors of somatostatin and can be regulated especially via somatostatin receptor subtype 2 (SSTR_2_) [27]. Sct induces elevation of cAMP levels and cell proliferation in cholangiocytes as mentioned, and somatostatin abolishes these effects and inhibits cholangiocyte proliferation [28]. A previous study using seven CCA tumor tissues found that all CCA tumors expressed mRNA of SSTR_2_ [29]. A somatostatin analogue, octreotide, inhibited cell proliferation of human CCA line SK-ChA-1 cells, and another analogue, lanreotide, decreased tumor weights in SK-ChA-1 xenograft mice, indicating the potentials of somatostatin or its analogues as therapeutic drugs for CCA [29]. Analysis of mRNA expression for SSTR isoforms (subtype 1 to 5) in CCA cell lines, QBC939, RBE, NEC, and SSP25, detected only SSTR_2_ in these cell lines [30]. Octreotide administration into QBC939 xenograft mice decreased the tumor size [30]. However, a study using 20 patients with CCA or gallbladder adenocarcinoma found that administration of lanreotide had minimal therapeutic effects with no significant improvement of tumor conditions, regardless of the detection of SSTR in patients by radiolabeled somatostatin analogues [31]. In addition, histological analysis using 27 CCA tumor tissues detected SSTR_2_ from only 30% of CCA tumors [32]. This study identified other SSTR isoforms from CCA tissues; SSTR_1_ was detected from 67% of CCA tumors, SSTR_5_ was from 11%, and SSTR_3_ and SSTR_4_ were not detected [32]. Although administration of somatostatin or its analogues may be therapeutic to inhibit CCA progression, further studies are required to evaluate their effects in human patients, and responses against somatostatin treatments may differ depending on CCA phenotypes with different expression levels of SSTR and its subtypes in the tumor.

#### 2.1.3. Melatonin

Melatonin is a hormone secreted predominantly in the pineal gland from serotonin. Serotonin is converted to melatonin by aralkylamine *N*-acetyltransferase (AANAT) and acetyl serotonin *O*-methyltransferase (ASMT) [33]. Cholangiocytes express AANAT and ASMT as well as melatonin receptors MT_1_ and MT_2_, indicating the local melatonin production and signaling in the liver [34]. Melatonin has inhibitory effects on cholangiocytes, and melatonin administration or overexpression of AANAT decreased cAMP levels and VEGF expression in cholangiocytes, leading to decreased ductular reaction in BDL rats in vivo [35,36,37]. Melatonin administration also decreased ductular reaction and liver fibrosis in *Mdr2*^-/-^ mice by inhibiting cholangiocyte proliferation and HSC fibrogenesis, suggesting that melatonin can be utilized as a therapeutic drug for PSC [38]. A previous study has demonstrated that expression levels of AANAT and ASMT are downregulated in human CCA line Mz-ChA-1 cells compared to human normal cholangiocyte line H69 cells, and melatonin treatments inhibited Mz-ChA-1 cell proliferation and decreased tumor size in xenograft mice by inducing apoptosis in CCA cells, supporting the melatonin-mediated autocrine regulation of cholangiocytes or CCA cells [39]. Another study also demonstrated the inhibitory effects of melatonin on CCA cell proliferation using KKU-M055 and KKU-M214 cells [40]. Infection with liver flukes, such as *Opisthorchis viverrini* and *Clonorchis sinensis*, is a common risk factor for CCA development in Asia [4]. Liver fluke infestation was utilized with the combination of carcinogen administration, *N*-nitrosodimethylamine, to induce CCA tumors in hamsters [41]. In this model, melatonin administration decreased CCA tumor size and improved survival rates of affected hamsters by inhibiting apoptosis and restoring functions of mitochondria [41]. CCA tumors are often accompanied by dense stromal tissues including immune cells. Melatonin administration decreased the population of CD4^+^, IL-17^+^, and FOXP3^+^ cells in the liver, which probably leads to the inhibition of CCA tumor growth in this hamster model [42]. These studies suggest the promising therapeutic effects of melatonin against CCA; however, it is undefined whether melatonin can be effective on all CCA phenotypes since CCA tumors may have different expression levels of melatonin receptors, and responses against melatonin may differ depending on CCA tumors with different origins.

#### 2.1.4. Estrogen

Estrogen is a primary female sex hormone produced mainly in ovaries and binds to two types of estrogen receptors, ER-α and ER-β [43]. Cholangiocytes express both ER-α and ER-β, but at normal conditions, expression levels of these receptors are barely detectable [44,45]. Interestingly, expression levels of both ER-α and ER-β are upregulated at diseased conditions including PSC and alcoholic cirrhosis [44]. BDL elevated ER-α and ER-β expression in cholangiocytes in vivo, and treatment with 17β-estradiol, a type of estrogen, induced cell proliferation of primary cholangiocytes isolated from normal rats [45]. CCA tumors had high levels of ER-α and ER-β detected by immunohistochemistry, indicating the regulations of cholangiocytes and CCA cells by estrogen [44]. A previous study has demonstrated that 17β-estradiol induces cell proliferation and decreases apoptosis in human CCA line HuH-28 cells [46]. Another study found that CCA tumors expressed high levels of VEGF-A and VEGF-C, and 17β-estradiol elevated expression levels of these VEGFs and their receptors in HuH-28 cells leading to increased cell proliferation [47]. Glyphosate, which is a common herbicide used worldwide, has been reported to activate ER-α leading to elevated cell proliferation under unknown mechanisms [48]. Stimulation of HuCCT1 cells, which express both ER-α and ER-β, with 17β-estradiol or glyphosate at the same concentrations increased cell proliferation by activating the ERK1/2 pathway [49]. Administration of 17β-estradiol induced cell proliferation in human CCA line KKU-213 and KKU-139 cells and increased tumor weights in xenograft mice with either of these CCA cells [50]. In fact, administration of an estrogen receptor modulator, tamoxifen, abolished these proliferative effects of estrogen [50]. Therefore, these studies suggest that estrogens and activation of their receptors induce CCA tumor growth.

Since estrogens are female hormones, these results raise a question whether females are more susceptible to CCA than males. A statistical analysis has shown that the incidence of intrahepatic CCA in the US is increasing in both males and females, and males have higher incidence than females [51]. Another study analyzed CCA mortality in the US and found that females had lower mortality rates compared to males, indicating that higher estrogen levels in women do not increase the susceptibility of CCA formation or progression [52]. Functions of estrogen receptors may differ between ER-α and ER-β. Thioacetamide (TAA) is a carcinogen which induces liver fibrosis, cirrhosis, and CCA [53,54]. Administration of KB9520, a selective ER-β agonist, decreased cell proliferation and increased apoptosis in HuH-28 cells in vitro [55]. KB9520 also inhibited CCA tumor formation as well as tumor growth after tumor formation in TAA-induced CCA rat models [55]. Genistein is another ER-β agonist, and treatments with genistein inhibited cell proliferation of human intrahepatic CCA lines (HuCCT1 and RMCCA-1) by decreasing activation of AKT and ERK1/2 signaling pathways, indicating that activation of ER-α or ER-β results in different effects in CCA cells [56]. In addition, CCA may change estrogen production and secretion regardless of gender. A previous study analyzed serum samples from 54 male CCA patients and 68 male healthy individuals and found that CCA patients had significantly higher serum estrogen levels than control subjects, and these high levels of estrogen were correlated with poor survival rates [57]. Aromatase (CYP19A1) is a key enzyme for the production of estrogens. A study using CCA tumor tissues from 51 males and 23 females found that CCA tumors had elevated expression of aromatase, and high aromatase expression was correlated with poor survival rates in males but not in females [58]. CCA line KKU-100 and KKU-213 cells also express elevated levels of aromatase compared to normal cholangiocytes, and inhibition of aromatase expression by siRNAs decreased cell proliferation and migration in KKU-213 cells [58]. Although the inhibition of estrogen production especially in males may be a promising therapeutic approach, further studies are required to elucidate detailed mechanisms and functional roles of local estrogen production in the liver and activation of signaling pathways via ER-α or ER-β receptors.

#### 2.1.5. Insulin-Like Growth Factor

Insulin-like growth factor (IGF) is a hormone which has high similarity to insulin and may be associated with cancer development [59]. There are two types of IGF, IGF-1 and IGF-2, and corresponding receptors, IGF-1R and IGF-2R. RT-PCR detected expression of both IGF-1R and IGF-2R in human CCA lines including HuH-28 and TFK-1 cells [60]. Immunohistochemistry detected elevated expression levels of IGF-1 and IGF-1R in CCA tumors compared to normal liver tissues [46]. IGF-1 treatments induced cell proliferation of HuH-28 cells by elevated activation of AKT and ERK1/2 signaling pathways [46]. CCA cells often express elevated levels of epidermal growth factor receptor (EGFR). IGF-1, EGF, and estradiol induce cancer cell adhesion via IGF1R and activation of ERK1/2 [61]. Tyrosine kinase inhibitors, such as erlotinib, inhibit EGFR activation and have anti-cancer effects, although CCA tumors often have resistance to EGFR inhibitors [62]. Vaquero et al. generated erlotinib-resistant CCA cells by escalating exposure to erlotinib from 1 to 20 µM and found that resistant CCA lines, including Mz-ChA-1, HuCCT1, and SK-ChA-1 cells, expressed higher levels of IGF-2 and IGF-1R as well as insulin receptor (IR) [63]. IR/IGF-1R inhibitor, linsitinib, decreased tumor volumes in xenograft mice with erlotinib-resistant CCA tumors [63]. A study analyzing serum and bile samples of 29 patients with extrahepatic CCA, 19 patients with pancreatic cancer, and 25 patients with benign biliary abnormalities found that CCA patients have significantly elevated levels of IGF-1 in bile compared to other patient groups but not in serum [64]. Another study using samples from 62 patients with biliary obstructions, such as PSC or chronic pancreatitis, and 47 patients with malignant biliary obstructions including CCA and head of pancreas tumor demonstrated that patients with malignant obstructions had increased levels of IGF-1 and VEGF in bile but not in serum [65]. Malignancies in the bile ducts are strongly associated with IGF in bile, and inhibition of IGF signaling may be a promising target to develop novel therapies for CCA.

#### 2.1.6. Gastrin

Gastrin is a gastrointestinal hormone that regulates gastric acid secretion [66]. Gastrin binds to the cholecystokinin B receptor (CCK-BR), and cholangiocytes express this receptor indicating the regulation of cholangiocyte functions by gastrin [67]. Sct induces secretion and proliferation of cholangiocytes by elevating cAMP levels, and gastrin abolishes these effects [67]. Administration of gastrin inhibited ductular reaction and cholangiocyte proliferation by decreasing cAMP levels in BDL rats in vivo, suggesting its inhibitory effects on cholangiocytes [68]. Histological analysis using 10 CCA tumor tissues identified elevated expression levels of CCK-BR and gastrin precursors in CCA tumors, indicating local gastrin production and signaling activation in CCA tumors [69]. CCA lines, Mz-ChA-1, HuH-28, and TFK-1 cells, expressed CCK-BR, and gastrin treatments decreased cell proliferation in CCA cells by inducing apoptosis via elevated inositol 1,4,5-triphosphate (IP_3_) secretion and protein kinase C alpha (PKC-α) expression [70]. Although these studies suggest the anti-cancer effects of gastrin against CCA, previous studies are limited, and detailed mechanisms are undefined.

### 2.2. Neuropeptides

#### 2.2.1. Nerve Growth Factor

NGF is a neuropeptide associated with cell growth and plays an important role with VEGF in cholangiocyte proliferation as mentioned [16]. During BDL, NGF expression is upregulated in cholangiocytes, leading to elevated ductular reaction and biliary inflammation in rodents [71,72]. CCA tumors express elevated levels of VEGF [47]. Immunohistochemistry for 28 hilar CCA tumor tissues identified elevated NGF-β expression in 57.1% of CCA cases, and robust NGF-β expression was positively correlated with elevated VEGF-C expression, indicating the association of NGF-β with CCA proliferation [73]. Another study has demonstrated that NGF-β is overexpressed in human CCA line QBC939 cells, and induces tumor growth in xenograft mice models [74]. NGF has two receptors, low affinity p75 neurotrophin receptor (p75NTR) and high affinity tropomyosin receptor kinase A (TrkA) [75]. A study using 83 intrahepatic CCA samples has demonstrated that NGF and TrkA are upregulated in CCA tumors, and high levels of NGF and TrkA expression are associated with poor survival rates of patients [76]. NGF-β treatments induced cell proliferation and invasion of human intrahepatic CCA line RBE cells [76]. However, immunohistochemistry for 112 extrahepatic CCA samples demonstrated that 55% of patients had high NGF expression and 45% of patients had low expression, and there was no significant association between NGF expression levels and survival rates [77]. In addition, previous studies showing the association between NGF and CCA have been reported form Asian countries, and a study analyzing 93 CCA tumor tissues from Caucasian patients has reported that NGF-β and all Trk isoforms (A, B, and C) could not be detected from these Caucasian samples, regardless of the location of CCA (intrahepatic, hilar, or distal) [78]. NGF-Trk signaling could be a therapeutic target for the novel CCA therapy, the efficacy may differ between races, and genetic traits may be involved in NGF-mediated CCA growth.

#### 2.2.2. Substance P

Substance P (SP) is a neuropeptide which binds to neurokinin 1 receptor (NK-1R) with high affinity [79]. BDL elevates serum levels of SP in rats [80], and mRNA expression levels of NK-1R and tachykinin precursor 1 (TAC1), which encodes the precursor of SP, are upregulated in liver samples of BDL mice, *Mdr2*^-/-^ mice, and late stage PSC patients [81]. Administration of SP caused elevated ductular reaction and liver fibrosis in C57BL/6 wild-type mice, and NK-1R^-/-^ mice had improved liver conditions after BDL compared to wild-type mice with decreased HSC activation and fibrogenesis, indicating the association between the SP/NK-1R axis and cholestatic liver injury [81]. Human CCA lines, Mz-ChA-1, SG231, HuH-28, CCLP, and HuCCT1 cells, express higher levels of TAC1 and NK-1R compared to human normal cholangiocytes [82]. SP increased cell proliferation of these CCA cell lines, and administration of NK-1R antagonist, L-733,060, decreased Mz-ChA-1 cell proliferation in vitro as well as tumor size in Mz-ChA-1 xenograft nude mice in vivo [82]. Although NK-1R antagonists may be therapeutic in inhibiting CCA tumor growth, current studies are limited and expression levels of TAC1 and NK-1R in human CCA tumor tissues and correlation with metastases or survival rates are still undefined.

#### 2.2.3. Neuropeptide Y

Neuropeptide Y (NPY) regulates various biological actions in the gastrointestinal tract via NPY receptors, and five subtypes of NPY receptors have been identified in mammals [83,84]. NPY is a physiological substrate of fibroblast activation protein, which is strongly associated with liver fibrosis via HSC activation [85]. A previous study has demonstrated that BDL elevated expression of NPY in the rat bile ducts [86]. Administration of NPY or anti-NPY antibody decreased or increased ductular reaction in BDL rats, respectively, indicating the inhibitory effects of NPY on cholangiocyte proliferation [86]. Immunohistochemistry identified elevated NPY levels in CCA tumor samples from 48 patients [87]. NPY treatments inhibited cell proliferation of CCA lines, Mz-ChA-1, SG231, HuH-28, CCLP, HuCCT1, and TFK-1 cells, via elevation of IP_3_ secretion and PKC-α activation [87]. Administration of NPY decreased tumor volume in Mz-ChA-1 xenograft mice, indicating promising anti-cancer effects against CCA, although current studies are limited and further experimental evidence is required [87].

### 2.3. Neurotransmitters

#### 2.3.1. Dopamine

Dopamine is a neurotransmitter, which plays an important role in the brain. Parkinson’s disease is a common neurological disorder in the elderly characterized by degeneration of dopaminergic neurons. Dopaminergic denervation is associated with liver malfunctions, indicating the regulations of liver functions by dopamine [88]. There are five subtypes of dopamine receptors identified to date, and BDL elevates expression levels of D_2_ dopamine receptor in rat cholangiocytes [89]. Treatments with an agonist for D_2_ and D_3_ receptor, quinelorane, inhibited upregulation of cAMP levels and PKA activity induced by Sct in cholangiocytes [89]. Since elevated cAMP and PKA activity induces proliferation and secretion of cholangiocytes [90], this study suggests that dopamine has the inhibitory effects on cholangiocyte proliferation and functions. Dopamine is converted from tyrosine by enzymes tyrosine hydroxylase (TH) and dopa decarboxylase (DDC), and a previous study found that CCA tumors had higher expression levels of TH and DDC compared to normal liver tissues detected by immunohistochemistry using 48 CCA tumor tissues [91]. Human CCA lines, Mz-ChA-1, SG231, CCLP, and HuCCT1 cells, had significantly higher levels of dopamine secretion as well as TH and DDC expression compared to normal cholangiocytes [91]. Dopamine treatments induced CCA cell line proliferation, and administration of a specific DDC inhibitor, L-(-)-α-methyldopa, decreased tumor volumes in Mz-ChA-1 xenograft mice in vivo [91]. Although inhibition of local dopamine production in CCA tumors could be a therapeutic approach, current studies are limited, and further studies are needed to evaluate the efficacy of TH or DDC inhibitors as drugs for CCA.

#### 2.3.2. Serotonin

Serotonin (5-hydroxytryptamine, 5-HT) is a neurotransmitter associated with sleeping, mood and depression, eating and digestion, and cancer development [92]. Serotonin is synthesized from tryptophan by tryptophan hydroxylase (TPH), and degraded by monoamine oxidase (MAO) [93]. TPH1^-/-^ mice had higher liver damage and necrosis after BDL compared to wild-type mice via elevated bile salt secretion, indicating the inhibitory effects of serotonin on cholangiocyte proliferation and secretion [94]. There are seven families of serotonin receptors with a number of subtypes, which have different functions and characteristics [93]. Cholangiocytes express serotonin receptors 5-HT_1A_ and 5-HT_1B_ during BDL, and administration of serotonin receptor agonists (8-OH-DPAT for 5-HT_1A_ and anpirtoline for 5-HT_1B_) decreased BDL-induced ductular reaction and cholangiocyte proliferation by inhibiting elevation of cAMP and PKA activity [95]. However, a recent study has demonstrated that cholangiocytes express other receptor subtypes, 5-HT_2A_, 5-HT_2B_, and 5-HT_2C_, and agonists for these receptors exacerbated ductular reaction and liver fibrosis in BDL rats [96]. Antagonists of these receptors attenuated BDL-induced liver damage in vivo, indicating that activation of serotonin receptors affects liver conditions differently depending on the receptor [96]. Immunohistochemistry for 48 samples from CCA patients identified elevated expression levels of TPH1 and decreased levels of MAO-A in CCA tumors [97]. CCA patients had higher serotonin levels in bile compared to control individuals, and human CCA cell lines, Mz-ChA-1, SG231, HuH-28, CCLP, HuCCT1, and TFK-1 cells, also secrete higher levels of serotonin compared to normal cholangiocyte H69 cells [97]. Serotonin treatments induced cell proliferation in CCA cell lines in vitro, and administration of a specific TPH1 inhibitor, *p*-chlorophenylalanine, decreased tumor volume in Mz-ChA-1 xenograft mice in vivo [97]. A previous study analyzed intra-platelet serotonin levels in 96 patients with liver cancers, including colorectal cancer liver metastasis, HCC, and CCA, and found that high serotonin levels were associated with increased incidence of postoperative disease recurrence, indicating the promotion of cancer growth by serotonin [98]. Another study using 43 intrahepatic CCA and 84 hilar CCA tumor tissues found that expression levels of MAO-A were significantly downregulated in CCA tumors compared to 45 benign tissues [99]. No or low MAO-A expression was correlated with poor survival rates of CCA patients [99]. Overexpression of MAO-A in Mz-ChA-1 cells decreased cell proliferation as well as tumor volumes in xenograft mice [99]. Although inhibition of TPH1 to decrease serotonin production or expression of MAO-A to promote serotonin degradation may be a promising therapeutic approach for CCA, functional roles of serotonin receptors may differ depending on the subtype, and further studies are required to elucidate the detailed mechanisms.

#### 2.3.3. Histamine

Histamine is a mediator, which is predominantly secreted from mast cells and associated with allergic and inflammatory responses via interaction with four subtypes of histamine receptors (H_1_-H_4_) [100]. Previous studies have demonstrated that serum histamine levels are upregulated in BDL rats or *Mdr2*^-/-^ mice, and administration of cromolyn sodium, which stabilizes mast cells and inhibits histamine secretion, attenuates ductular reaction and liver fibrosis in those animals, indicating that histamine promotes cholangiocyte proliferation [101,102]. Histamine is converted from histidine by histidine decarboxylase (HDC) and degraded by MAO-B, and immunohistochemistry for 48 CCA tissues identified higher expression levels of histamine, HDC, and MAO-B in CCA tumors compared to normal tissues [103]. Human CCA cell lines secreted higher levels of histamine than normal cholangiocytes, and histamine increased tumor volumes in xenograft mouse models via elevation of VEGF-A and VEGF-C in Mz-ChA-1 cells [103]. Administration of cromolyn sodium decreased tumor size in Mz-ChA-1 xenograft mice, indicating that histamine promotes CCA tumor growth [104]. CCA cells express H_1_ and H_2_ receptors, and administration of antagonists for H_1_ receptor (mepyramine) or H_2_ receptor (ranitidine) decreased Mz-ChA-1 tumor size by decreasing expression levels of HDC and VEGF-A in the xenograft nude mouse model in vivo [105]. These results suggest that inhibition of histamine-H_1_/H_2_ signaling has anti-cancer effects to inhibit CCA tumor growth. However, a previous study has demonstrated that CCA tumors and cell lines express elevated levels of H_3_ receptor compared to normal tissues or cholangiocytes, and an agonist of H_3_ receptor (RAMH) inhibited cell proliferation of CCA cells as well as tumor growth in xenograft mice [106]. RAMH treatments induced PKC-α activation leading to apoptosis and decreased ERK1/2 activation leading to attenuated cell proliferation in Mz-ChA-1 cells [106]. Another study has demonstrated that CCA tumors and cell lines also express high levels of H_4_ receptor, and a H_4_ receptor agonist, clobenpropit, decreases CCA cell proliferation as well as tumor volumes in Mz-ChA-1 xenograft mice by inhibiting epithelial-mesenchymal transition of CCA cells [107]. Although histamine and its receptors are potential therapeutic targets, functional roles of histamine receptors may differ between H_1_/H_2_ and H_3_/H_4_ receptors, and effects of histamine may be different depending on the balance of receptor subtypes in the individual CCA tumors.

## 3. Conclusions and Future Perspectives

**Figure 1:**
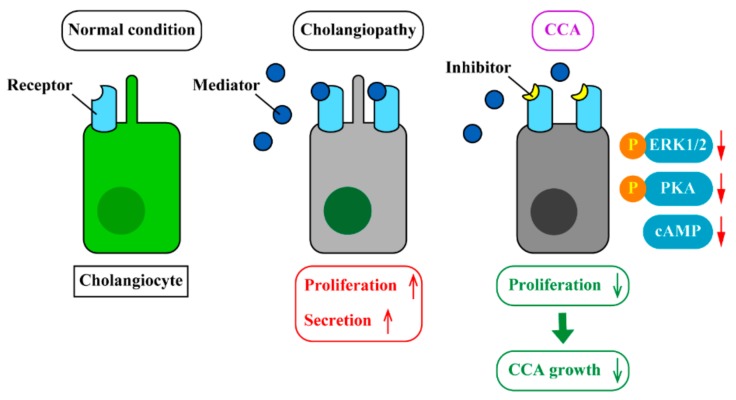
Strategy for the development of novel CCA therapies. Cholangiocytes express various receptors that interact with neuroendocrine mediators. During cholangiopathies such as primary sclerosing cholangitis (PSC), expression levels of mediators and receptors are upregulated, which induces cholangiocyte proliferation and secretion leading to ductular reaction and liver fibrosis. This alteration of cholangiocyte functions may cause CCA development. Upregulation of these mediators and receptors is also observed in CCA tumors. Inhibition of the mediator/receptor axis using antagonists for receptors decreases cAMP levels as well as activation of PKA/ERK1/2 pathways, which attenuates CCA progression and migration. However, effects of mediators may differ between cancerous and non-cancerous cholangiocytes depending on the mediators. In addition, cholangiocytes may express multiple subtypes of receptors for the specific mediators, and functions of receptors may differ between different subtypes. Further studies are required to develop a novel drug to target the specific mediator or receptor subtype for the regulations of CCA cell functions.

Previous studies have identified a number of neuroendocrine mediators associated with CCA progression. Table 1 lists these mediators and compares functional roles in CCA. Many mediators were found in PSC patients showing the association with ductular reaction and cholangiocyte proliferation, which were also related to the regulation of CCA progression and metastases. However, some mediators have different effects in PSC and CCA. For example, the Sct/SR axis is a well-studied signaling pathway in animal models and its activation promotes cholangiocyte proliferation and cytokine secretion leading to ductular reaction and liver fibrosis [108]. Although it is clear that Sct induces cholangiocyte proliferation, a contrary effect, inhibition of CCA proliferation, has been reported [25]. The same inconsistency was found in studies related to dopamine. Dopamine inhibited Sct-induced cAMP elevation in cholangiocytes but promoted proliferation of CCA cell lines [89,91]. These findings suggest that functional effects of mediators may differ when cholangiocytes become cancerous. CCA tumors can be derived from other cell types, and mediators may affect differently in different CCA phenotypes. In addition, there are multiple receptors for some mediators, and the effects of the mediator can differ depending on the families or subtypes of the receptor. For example, activation of estrogen receptor ER-α promotes CCA proliferation, but activation of ER-β inhibits CCA growth [46,47,55,56]. Although current studies have demonstrated promising therapeutic effects targeting the mediator and its receptor signaling pathways, further studies are required to understand differences in the efficacy of drugs targeting these pathways depending on the origins of CCA tumors and subtypes of receptors. Figure 1 summarizes a strategy for the development of novel CCA therapies targeting associated signaling pathways.

In conclusion, CCA tumors have altered expression levels of neuroendocrine mediators and their receptors, and targeting these signaling pathways may be a promising approach to establish novel treatments for CCA.

## Figures and Tables

**Table 1:** Comparison of neuroendocrine mediators associated with cholangiocarcinoma (CCA).

Mediator/Receptor	Upregulated/Downregulated in CCA	Promote/Inhibit CCA Growth	Note
**Hormones**			
Sct/SR	Positive [24]	Inhibit [25]	Only limited numbers of CCA are positive to SR [24]
Somatostatin/SSTR_2_	Positive [29]	Inhibit [29,30]	May not be effective in human CCA patients [31]
Melatonin/MT_1_ and MT_2_	Downregulated [39]	Inhibit [39,40]	May also inhibit immune cell infiltration [42]
Estrogen/ER-α and ER-β	Upregulated [44]	Promote [46,47]/inhibit [55,56]	Different functions between ER-α and ER-β [46,47,55,56]
IGF-1 and IGF-2/IGF-1R and IGF-2R	Upregulated [46]	Promote [46]	Could be utilized as biomarkers [64,65]
Gastrin/CCK-BR	Upregulated [69]	Inhibit [70]	Calcium-dependent [70]
**Neuropeptides**			
NGF-β/TrkA	Upregulated [73,76]	Promote [74,76]	May be limited in Asian patients [78]
SP/NK-1R	Upregulated [82]	Promote [82]	Lacking evidence in human CCA tumors
NPY/NPY receptors	Upregulated [87]	Inhibit [87]	Limited previous studies
**Neurotransmitters**			
Dopamine/dopamine receptors	Upregulated [91]	Promote [91]	Limited previous studies
Serotonin/5-HT receptors	Upregulated [97]	Promote [97]	Functions may differ between receptors [95,96,97]
Histamine/histamine receptors	Upregulated [103]	Promote [103,104,105] inhibit [106,107]	Functions differ between H_1_/H_2_ and H_3_/H_4_ receptors [103,104,105,106,107]

## Abbreviations

5-HT	5-hydroxytryptamine
AANAT	*N*-acetyltransferase
ASMT	acetyl serotonin *O*-methyltransferase
BDL	bile duct ligation
CCA	cholangiocarcinoma
CCK-BR	cholecystokinin B receptor
CK	cytokeratin
EGFR	epidermal growth factor receptor
HCC	hepatocellular carcinoma
HSCs	hepatic stellate cells
IGF	insulin-like growth factor
IL	interleukin
IR	insulin receptor
IP_3_	inositol 1,4,5-triphosphate
MAO	monoamine oxidase
Mdr2	multidrug resistance protein 2
NGF	nerve growth factor
NK-1R	neurokinin 1 receptor
p75NTR	p75 neurotrophin receptor
PKC-α	protein kinase C alpha
PSC	primary sclerosing cholangitis
Sct	ecretin
SP	substance P
SR	secretin receptor
SSTR_2_	somatostatin receptor subtype 2
TAA	thioacetamide
TAC1	tachykinin precursor 1
TrkA	tropomyosin receptor kinase A
TPH	tryptophan hydroxylase
VEGF	vascular endothelial growth factor
